# Managing Transcatheter Aortic Valve Failure

**DOI:** 10.1016/j.shj.2026.100806

**Published:** 2026-01-29

**Authors:** Daniel J. Blackman, Noman Ali, Annette Maznyczka, Mariama Akodad, David Hildick-Smith, Edoardo Zancanaro, Hendrik Treede

**Affiliations:** a Department of Cardiology, Leeds Teaching Hospitals, Leeds, UK; b Leeds Institute of Cardiovascular & Metabolic Medicine, University of Leeds, Leeds, UK; c Ramsay Générale de Santé, Institut Cardiovasculaire Paris Sud, Massy, France; d Department of Cardiology, Royal Sussex County Hospital, Brighton, UK; e Department of Cardiac Surgery, University Medical Center of the Johannes Gutenberg University, Mainz, Germany; f Division of Cardiac Surgery, Department of Surgery, Brigham and Women's Hospital, Harvard Medical School, Boston, Massachusetts, USA

**Keywords:** Bioprosthetic valve failure, Structural valve deterioration, Surgical aortic valve replacement, Transcatheter aortic valve replacement

## Abstract

As transcatheter aortic valve replacement (TAVR) expands rapidly into younger low-risk cohorts with long life expectancy, increasing numbers of patients will outlive their valves and present with bioprosthetic valve failure requiring treatment. Age, comorbidities, and the complexity of surgical explantation will make redo TAVR the preferred treatment option in the vast majority of patients. However, redo TAVR poses specific anatomical challenges, including an elevated risk of coronary artery obstruction, the preservation of coronary access, and optimization of hemodynamic outcomes and long-term durability, though few data exist to guide clinicians in the optimal procedural approach. Meanwhile, surgical explantation and valve replacement will remain a vital option in specific groups, including those with endocarditis and with anatomical features that prohibit redo TAVR.

This review article provides a detailed exposition of the challenges of both transcatheter and surgical treatment of transcatheter aortic valve failure, recommended strategies for diagnosis, preprocedural planning and procedural execution to deliver safe and effective outcomes, and a summary of the current evidence base in this growing field.

## Highlights

• With the expansion of transcatheter aortic valve (TAV) replacement (TAVR) into younger patients, TAV failure is an increasingly common clinical challenge.
• The diagnosis of TAV failure may be challenging; this review summarizes the multimodality approach required.
• Redo TAVR poses specific challenges to the operator, particularly in relation to coronary access and obstruction, and optimization of hemodynamics and subsequent durability. A step-by-step approach to preprocedural planning and effective procedural execution is described.
• Surgical TAV explant retains a key role in patients with TAV endocarditis and anatomical contraindications to redo TAVR; this review details the precise surgical techniques required for optimal outcomes.
• A comprehensive algorithm is presented for the overall approach to the diagnosis and management of TAV failure, both transcatheter and surgical, and for all failure mechanisms.

## Graphical abstract

Algorithm for the diagnosis and management of TAV failure. Abbreviations: AR, aortic regurgitation; BAV, balloon aortic valvuloplasty; BEV, balloon-expanded valve; BVF, bioprosthetic valve failure; CMR, cardovascular magnetic resonance; CRP, coronary risk plane; CT, computed tomography; EOA, effective orifice area; HALT, hypoattenuated leaflet thickening; N, no; NSP, neoskirt plane; NSVD, nonstructural valve dysfunction; OAC, oral anticoagulation; OMT, optimal medical therapy; PET, positron emission tomography; PPM, prosthesis–patient mismatch; PVR, paravalvular regurgitation; SAVR, surgical aortic valve replacement; SEV, self-expanding valve; SURPLUS, Surgical Resection of Prosthetic valve Leaflets Under Direct ViSion; SVD, structural valve deterioration; TAV, transcatheter aortic valve; TAVR, transcatheter aortic valve replacement; TEE, transesophageal echocardiogram; TTE, transthoracic echocardiogram; Y, yes.

**Figure:**
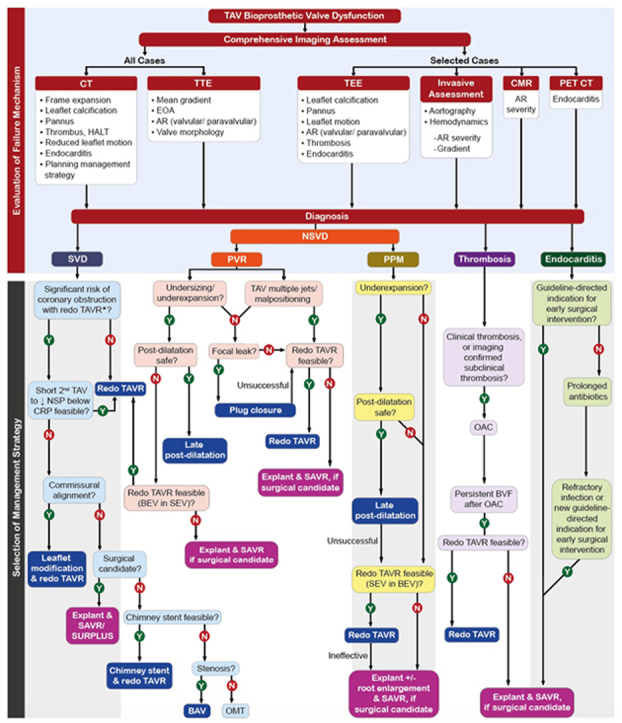

## Introduction

**Figure 1:**
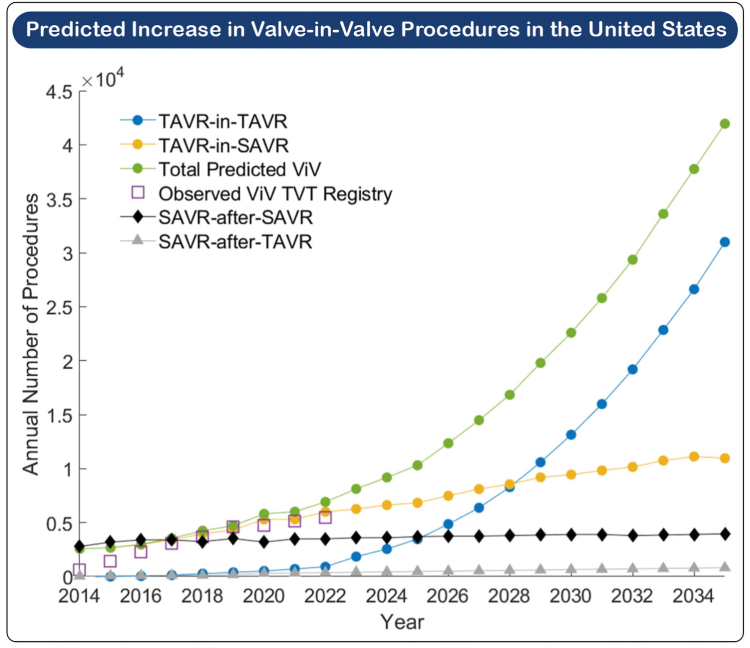
**Predicted increase in valve-in-valve procedures in the United States.** Reproduced from Généreux et al. Predicting treatment of bioprosthetic aortic valve failure in the United States: a proposed model. Struct Heart. 2024 Jul 10; 9(2):100,339. Abbreviations: SAVR, surgical aortic valve replacement; TAVR, transcatheter aortic valve replacement; TVT, Transcatheter Valve Therapies; ViV, valve-in-valve.

With annual procedure numbers in the United States exceeding surgical aortic valve replacement (SAVR) since 2019, transcatheter aortic valve (TAV) replacement (TAVR) has become the dominant treatment modality for severe aortic stenosis (AS).^1^^,^^2^ The current American and European guidelines for valvular heart disease support use of transfemoral TAVR in preference to surgery for patients ≥65 and ≥70 years of age, respectively, provided anatomical suitability.^3^^,^^4^ Recent years have seen a significant expansion in the use of TAVR in younger patient cohorts; 87.5% of patients aged between 65 and 80 and 47.5% of those <65 years of age who were treated for severe AS in the United States in 2021 underwent TAVR.^5^ Ongoing expansion of TAVR into younger patients with longer life expectancy will lead to an increasing number who outlive their TAV and will present with degeneration of the tissue valve, resulting in bioprosthetic valve failure (BVF). Although the incidence of reintervention for failed TAVR is currently low in representing only 0.28% of all TAVR procedures in the United States in 2023,^6^ it is forecast to accelerate rapidly. Using data from the Society of Thoracic Surgeons (STS)/American College of Cardiology (ACC) Transcatheter Valve Therapies registry, Genereux *et al*.^7^ have predicted that the number of redo TAVR procedures in the United States will reach 42,000 in 2035 and thereby represent 15% of all cases (Figure 1). As such, the management of failed TAVR will become an increasingly important facet of structural heart intervention.

## Mechanisms of TAV Failure

**Figure 2:**
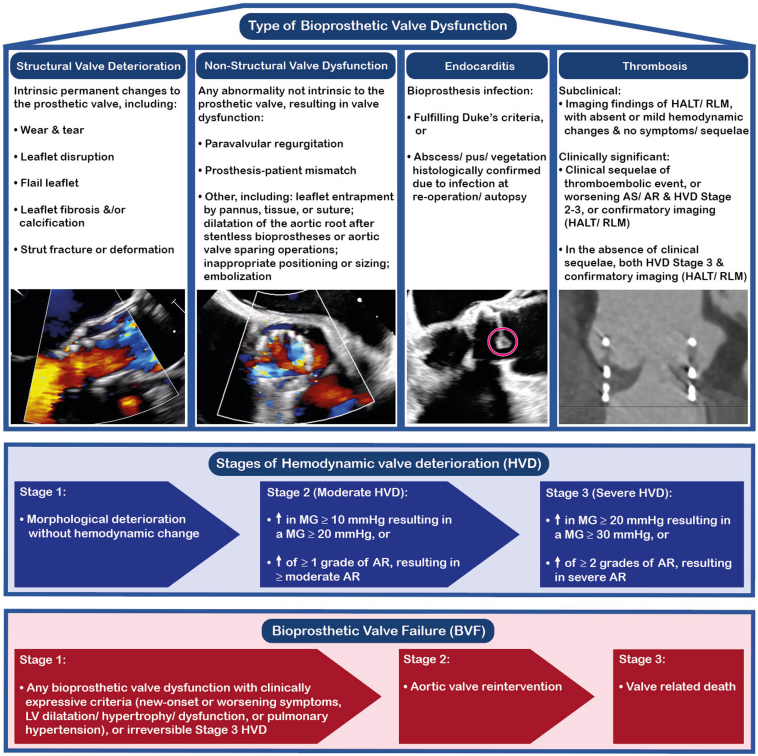
**Valve Academic Research Consortium (VARC)-3 definitions for bioprosthetic valve dysfunction and mechanisms of bioprosthetic valve failure.** Abbreviations: AR, aortic regurgitation; AS, aortic stenosis; HALT, hypoattenuated leaflet thickening; HVD, hemodynamic valve deterioration; LV, left ventricular; MG, mean gradient; PVL, paravalvular leak; RLM, reduced leaflet motion.

According to the Valve Academic Research Consortium (VARC)-3 definitions, BVF comprises bioprosthetic valve dysfunction (BVD) associated with a combination of hemodynamic change and clinical consequence (Figure 2). BVD can be categorized into the following four groups: 1) structural valve deterioration (SVD), 2) non-structural valve dysfunction (NSVD), 3) endocarditis, and 4) thrombosis.^8^

SVD refers to intrinsic changes in the valve such as leaflet fibrosis and/or calcification, flail leaflet, or wear and tear. SVD can manifest as valvular stenosis, regurgitation, or both, and it is the most common cause of BVD in TAVs. NSVD represents dysfunction unrelated to structural damage of the leaflets, most commonly paravalvular regurgitation (PVR) or prosthesis–patient mismatch (PPM). Valve thrombosis is most frequently subclinical, but it is hypothesized to act as a trigger to leaflet calcification and lead to SVD.^9^ TAV endocarditis is the least frequent cause of TAV BVD but has a poor prognosis.^10^

## Diagnosis of TAV Failure

Accurate diagnosis of the mechanism and severity of TAV BVF is often challenging and frequently requires a multimodality approach. A comprehensive review of best practices for imaging of valve failure is provided by the recent Heart Valve Collaboratory report.^11^ The key diagnostic tools and their specific roles in the diagnosis of BVF are summarized in the Graphical Abstract.

### Echocardiography

Still the gold standard in the initial evaluation of patients with suspected TAV BVF, transthoracic echocardiography (TTE) enables noninvasive assessment of both morphological and hemodynamic function. In many cases, TTE, combined with clinical assessment, may be sufficient for accurate diagnosis. TTE diagnosis of TAV BVF should always be based on serial changes in echo parameters (rather than an isolated snapshot) and should include flow-independent measures—that is, dimensionless index and effective orifice area.

Transesophageal echocardiography is of specific value in identifying morphological changes that may be unclear on TTE, including pannus and thrombus; assessing leaflet motion to differentiate SVD and PPM; identifying whether regurgitation is valvular or paravalvular; and diagnosing and characterizing endocarditis.

### Cardiac Computed Tomography Angiography

Computed tomography (CT) is mandatory in planning treatment of TAV failure, but is also valuable in diagnosis. CT will accurately identify generalized or localized frame underexpansion that may contribute to PPM and PVR; can identify morphological changes seen with endocarditis, clinical valve thrombosis (CVT), hypoattenuated leaflet thickening (HALT), and pannus; and diagnose reduced leaflet motion (RLM) and leaflet calcification.

### Cardiovascular Magnetic Resonance

Cardiovascular magnetic resonance has specific utility in the assessment of aortic regurgitation (AR) severity, which is frequently challenging on echocardiography post-TAVR. A regurgitant fraction of 30% or greater post-TAVR is considered clinically significant.^12^

### Positron Emission Tomography CT

Although positron emission tomography imaging has shown potential to detect early microcalification and predict valve dysfunction, its principal utility is to detect endocarditis, for which it has a class 1 recommendation in the 2023 European Society of Cardiology endocarditis guidelines.^13^

### Invasive Assessment of TAV Failure

Discordance between echocardiographic and invasive pressure gradients post-TAVR is well described and may vary by TAV type. Invasive assessment should be considered in cases where there is a discrepancy between the echocardiographic findings and the clinical presentation, particularly in those scenarios where pressure loss recovery is common and can lead to an elevated echocardiographic gradient despite normal valve function, including with small and/or balloon-expanded transcatheter valves and high-flow states. Invasive assessment of AR using aortography and hemodynamic assessment may also occasionally be valuable where doubt persists.

## Transcatheter Management of Nonstructural Valve Dysfunction

### Paravalvular Regurgitation

Moderate or severe PVR has been shown to be associated with a more than two-fold increase in all-cause mortality,^14^ and even mild PVR may be associated with an increased risk of adverse outcomes.^15^ The three transcatheter options for treating significant PVR post-TAVR are balloon postdilatation, percutaneous plug closure, or redo TAVR. Selecting the most appropriate of these depends on the mechanism of PVR combined with a detailed assessment of the post-TAVR CT.

Where PVR is a result of TAV frame underexpansion or undersizing, balloon postdilatation can be effective. In a small registry study, Akodad *et al*.^16^ reported on 11 patients with at least moderate PVR post-TAVR in whom late balloon postdilatation reduced the PVR to ≤ mild in all cases with no adverse effects. However, where PVR is a result of failure of the TAV to seal around an area of focal, protuberant calcification, balloon dilatation can be hazardous due to the risk of annular rupture. In these scenarios, percutaneous plug closure can be considered.^17^ In a series of 24 patients, plug closure reduced PVR to ≤ mild in 89% of cases.^18^ If multiple regurgitant jets are present, balloon postdilatation is high-risk or ineffective, or PVR is due to suboptimal implantation depth of the index TAV, redo TAVR should be considered. Implantation of a second valve can resolve significant PVR by extending the sealing zone between the original prosthesis and the anatomy through the creation of a neoskirt from the index TAV’s displaced leaflets. In addition, if a balloon-expanded valve (BEV) is used, redo TAVR can further expand a malapposed or undersized index TAV frame. In a multicentre study of 50 patients treated for PVR with redo TAVR, PVR was reduced to ≤ mild in 92% of cases.^19^

A multicentre registry of 201 patients comparing the three transcatheter techniques for treating PVR found the proportion of patients with persisting ≥ moderate PVR after a corrective procedure was the lowest in patients after redo TAVR (9.9%), followed by balloon postdilation (21.9%) and plug closure (25.9%).^17^

### Prosthesis–Patient Mismatch

Severe PPM post-TAVR is associated with an increased risk of mortality.^20^ The two transcatheter options for treatment of PPM are balloon postdilatation or redo TAVR.

In the Akodad registry, 11 of the 30 patients had moderate or severe PPM, which was corrected by late balloon postdilatation in over half (6/11; 54.5%).^16^ However, this treatment is only effective when PPM is a consequence of TAV frame underexpansion. In patients with a large body surface area, the use of a small intra-annular BEV can cause significant PPM despite adequate frame expansion.^21^ In this setting, redo TAVR with implantation of a supra-annular self-expanding valve (SEV) within the BEV can provide a larger effective orifice area and thereby treat PPM.

## Transcatheter Management of Thrombosis and Endocarditis

### Transcatheter Aortic Valve Thrombosis

TAV thrombosis encompasses a spectrum ranging from subclinical leaflet thrombosis (SLT) to overt CVT. The former is relatively common with the reported incidence varying between 12%-38%,^22^ whereas the latter is rare with a reported incidence of 1.2%.^23^ The initial stage of SLT is HALT, characterized by thickening of the valve leaflets, which may exist with or without RLM. SLT is most frequently diagnosed incidentally by means of a CT scan that identifies HALT with or without RLM. Systematic CT evaluation post TAVR has shown that, independent of antithrombotic treatment, the presence of HALT in individual patients fluctuates over time.^24^ Given its dynamic nature and the lack of associated symptoms or hemodynamic consequence, the clinical significance of SLT is uncertain. In a prospective observational registry of 115 patients with HALT over a median follow-up of 3 years, no association was observed with mortality or cerebrovascular events.^25^ However, SLT may act as a trigger for SVD—multivariable analysis from the same study revealed HALT to be a predictor of SVD.

Since HALT and RLM can resolve spontaneously, the diagnosis of SLT does not necessitate a change in antithrombotic therapy. Close echocardiographic follow-up is recommended, and treatment should be initiated if SLT progresses to CVT, with development of symptoms or elevated transvalvular gradients. First-line therapy for patients established on single antiplatelet therapy is a switch to anticoagulation, with the use of either vitamin K antagonists (VKAs) or direct oral anticoagulants.^22^ For patients who have developed CVT while already on a direct oral anticoagulant, switching to a VKA is recommended. If CVT occurs or persists in a patient established on a VKA, a higher target INR can be used, possibly in conjunction with addition of single antiplatelet therapy. In cases where CVT results in profound hemodynamic compromise with cardiogenic shock, thrombolysis or valvular reintervention may be required.

### Endocarditis

With the incidence in observational registries varying between 0.3-2.0 per 100-person years, infective endocarditis (IE) following TAVR is rare.^26^ However, outcomes are poor with 1-year mortality ranging between 27%-75%. Prolonged duration intravenous antibiotics (≥6 weeks) are the mainstay of treatment, with surgical intervention according to conventional indications for prosthetic IE.^13^

## Transcatheter Management of Structural Valve Deterioration

Medical therapy is ineffective in the management of TAV BVF and should be reserved for patients considered unsuitable for further transcatheter intervention or surgery, whereas advanced age and/or comorbidities will preclude surgery in most patients. Redo TAVR will, therefore, be the preferred treatment for the majority of subjects presenting with BVF due to SVD. However, redo TAVR presents specific challenges that need to be considered and overcome if it is to offer a safe, effective, and durable solution.

### Coronary Obstruction

Implantation of a second TAV frame within the index TAV will displace the leaflets of the first valve, effectively creating a tube graft between the two valve frames (the neoskirt), which has the potential to compromise coronary blood flow either through direct coronary ostial occlusion or at the level of the sinotubular junction (STJ)—so-called sinus sequestration. An increased incidence of coronary obstruction from the deflected bioprosthetic leaflets during valve-in-valve TAVR for degenerated surgical bioprostheses is well described.^27^^,^^28^ The risk is likely to be higher still with redo TAVR, as the neoskirt will be taller and more likely to extend above the coronary ostia and even the STJ—particularly in tall-frame devices and those with supra-annular leaflets. Studies using CT simulation to model redo TAVR have suggested that STJ sequestration may occur in up to a quarter of patients, particularly when the index valve is a supra-annular device.^29^^,^^30^

### Compromised Coronary Access

Redo TAVR is also likely to compromise access to the coronary arteries through interference created both by the neoskirt and the double-frame structure, especially in cases with misaligned TAV cells and if the index and/or second TAV is an SEV. CT simulation indicates that coronary access may be compromised in up to 78% of patients.^29^

### Hemodynamic Function and Long-Term Durability

Implantation of one valve frame within another will inevitably lead to smaller internal dimensions for the second TAV. Undersizing of the second TAV relative to the index device may be required while incomplete and eccentric expansion are also likely to be more frequent. Not only will this translate to compromised acute hemodynamic function with elevated transvalvular gradients, but underexpansion and eccentric expansion can also lead to pinwheeling, central regurgitation, PVR, and valve thrombosis—all of which may contribute to reduced valve durability.^31^^,^^32^

## Approach to Redo TAVR Procedures

### Preprocedural Planning

**Figure 3:**
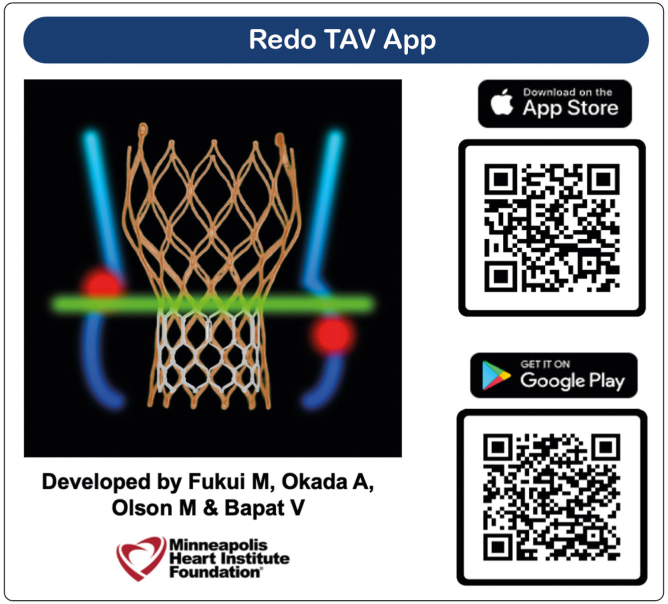
**Redo TAV App with QR code.** Abbreviation: TAV, transcatheter aortic valve.

**Figure 4:**
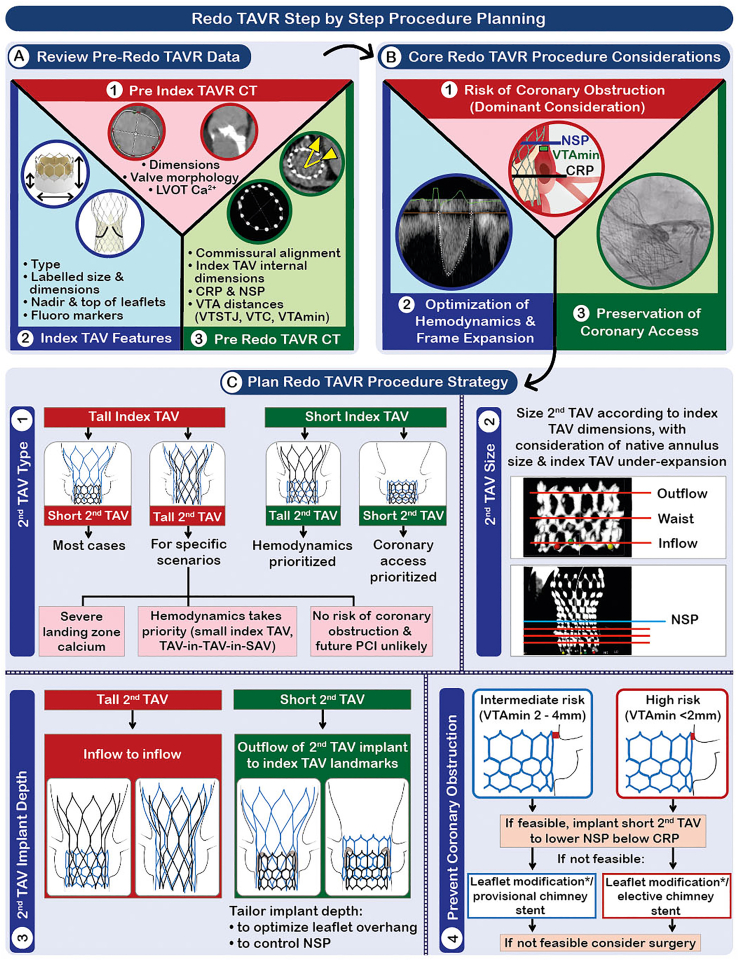
**Redo TAVR step-by-step procedure planning.** Leaflet modification feasible only if ≤ mild coronary misalignment. Abbreviations: Ao, aorta; Ca^2+^, calcium; CRP, coronary risk plane; CT, computed tomography; LVOT, left ventricular outflow tract; NSP, neoskirt plane; PCI, percutaneous coronary intervention; SAV, surgical aortic valve; SEV, self-expanding valve; TAVR, transcatheter aortic valve replacement; VTA, valve to aorta; VTA_min_, minimum valve to aorta; VTC, valve to coronary; VTSTJ, valve to sinotubular junction.

Preprocedural planning with a focus on CT analysis is perhaps of even greater importance in redo procedures. A wide variety of resources are already available to guide operators, including a comprehensive review by Bapat *et al*.^33^ and the Redo TAV app (Figure 3), which provides details of the technical characteristics of different transcatheter valves together with a step-by-step guide to CT analysis and preprocedural planning. Figure 4 summarizes the key steps to plan a safe and effective procedural strategy for redo TAVR.

Redo TAVR planning should incorporate analysis of the following data sources.

#### The Preindex TAVR CT

If available, the preindex TAVR CT should be reviewed to understand the native valve anatomy, specifically valve morphology (bicuspid/tricuspid); the presence of landing zone calcification; the original dimensions of the annulus that may impact redo TAV size selection; and the sinus of Valsalva and STJ dimensions together with the coronary heights, which will give an indication of the potential risk of coronary compromise with redo TAVR.

#### The Index TAV

The type and size should be identified and the key characteristics understood—specifically, the frame height; internal diameters at different levels; and, most importantly, the levels of the nadir and top of the leaflets, which will determine the potential neoskirt plane (NSP).

#### The Pre-Redo TAVR CT

**Figure 5:**
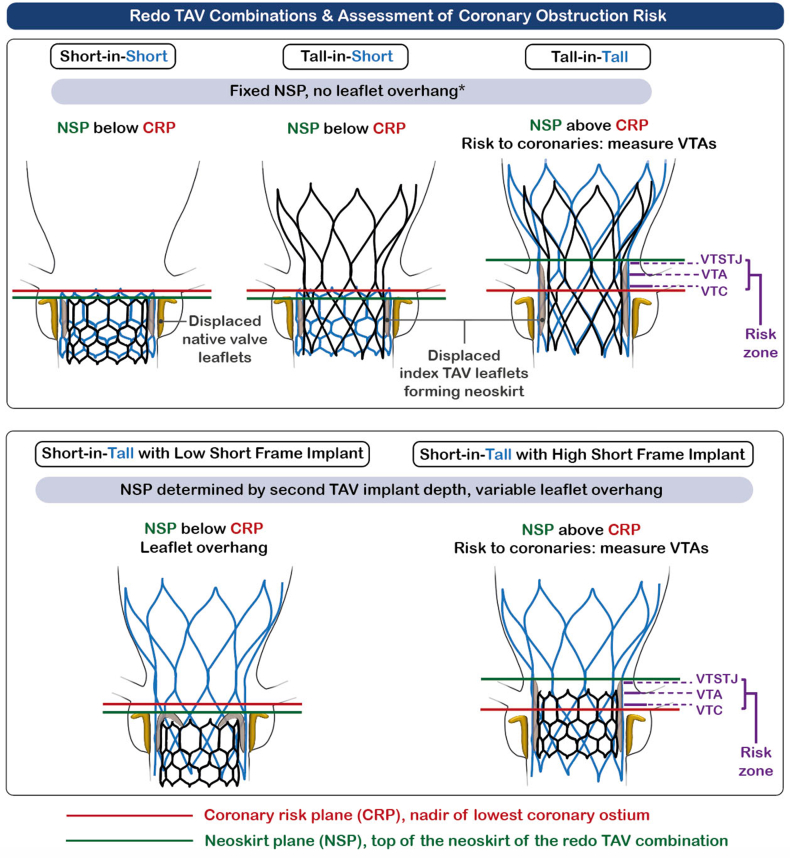
**Redo TAV combinations and assessment of coronary obstruction risk.** In some short-in-short cases, the second TAV may be implanted deeper to lower the NSP below the CRP with consequent leaflet overhang. Abbreviations: CRP, coronary risk plane; NSP, neoskirt plane; TAV, transcatheter aortic valve; VTA, valve to aorta; VTC, valve to coronary; VTSTJ, valve to sinotubular junction.
iv. Measurement of the NSP, defined as the plane at the top of the neoskirt created by the displaced index TAV leaflets and dependent on both the redo TAV combination and the intended implant depth of the second TAV. With tall-in-tall or tall-in-short redo TAV combinations, the NSP will be fixed and determined by the top of the index TAV leaflets. For short-in-tall and potentially for short-in-short, the NSP will vary depending on the implantation height of the second TAV. If the second TAV is implanted with its frame outflow below the top of the index TAV leaflets, then leaflet overhang will be created and can be used to mitigate the risk of coronary obstruction and to help preserve coronary access.
v. Assessment of the NSP in relation to the CRP and STJ. If the NSP is above the CRP, then there is a potential risk of coronary obstruction. If the NSP is above the STJ, there is a risk of sinus sequestration.
vi. Measurement of the valve-to-aorta (VTA) distances. If the NSP is above the CRP, then the distance between the created neoskirt and the coronary ostia and aortic sinus wall should be measured to further determine the risk of coronary obstruction. The VTA should be measured at multiple levels between the NSP and CRP, including the valve-to-coronary (VTC) distance; the minimum VTA (VTA_min_) distance; and, if the NSP is above the STJ, the valve-to-STJ (VTSTJ) distance. In most cases, the VTA is measured from the index TAV to the aorta. However, if the second TAV is balloon-expandable and has the potential to overexpand the index TAV, then the measurements should be made from a circle equivalent to the diameter of the second TAV.

CT analysis of a failing TAV requires a specific tailored step-by-step approach. Some TAVR CT analysis packages, including 3mensio (Pie Medical Imaging, Utrecht, Netherlands) have a dedicated redo TAVR workstream, while the Redo TAV app offers a detailed and highly comprehensive step-by-step guide. The key steps are as follows:

i. Assessment of commissural and coronary alignment. This will determine the feasibility of leaflet modification.
ii. Internal dimensions of the index TAV. Measurements should be undertaken at multiple levels according to the index TAV type and planned second TAV.
iii. Coronary ostia and the coronary risk plane (CRP). The position of the bottom of both coronary ostia in relation to the index TAV should be determined and defined by both their height in mm above the index TAV inflow and their position in relation to the index TAV landmarks that delineate the position of the leaflets (e.g. the nodes [Corevalve/Evolut (Medtronic, Minneapolis, Minnesota), Portico/Navitor (Abbott Vascular, Santa Clara, California)], upper crowns [Acurate; Boston Scientific, Minneapolis, Minnesota], and commissure tabs [SAPIEN; Edwards Lifesciences, Irvine, California]). The CRP is defined as the nadir of the lowest coronary ostium (Figure 5).

### Procedural Strategy

Four key decisions are required in every redo TAVR procedure:

1. Second TAV type
2. Second TAV size
3. Implant depth
4. The need for coronary protection and/or leaflet modification

These decisions will be dependent on the index TAV and planned second TAV type and should be made with focus on the three key considerations for all redo TAVR procedures:

1. Avoiding coronary obstruction—which should be considered primary
2. Preserving coronary access where possible
3. Optimizing hemodynamics and durability

#### Second TAV Type

##### Tall Index TAV

A short second TAV (short-in-tall) is favored in most cases to allow mitigation of the risk of coronary obstruction through control of the NSP and facilitate preservation of coronary access. However, tall-in-tall may be considered if optimization of hemodynamics is a priority (e.g., small index TAV, index TAV-in-surgical aortic valve [SAV]) or if landing zone calcification makes use of a BEV hazardous, but only if the risk of coronary obstruction is low and future coronary access is unlikely to be required.

##### Short Index TAV

Either a short or tall second TAV can be considered. The NSP—and thus the risk of coronary obstruction—will be constant regardless of the second TAV type in most cases. Tall-in-short will provide superior hemodynamics, whereas short-in-short can facilitate coronary access and may allow reduction of NSP height with a deeper implant in selected cases where coronary obstruction is a concern.

#### Second TAV Size

Sizing should be based primarily on the measured internal dimensions of the index TAV within the planned implant position of the second TAV. As per usual practice, area- and perimeter-based sizing are used with BEVs and SEVs, respectively. However, reference should also be made to the native annulus dimensions and to potential underexpansion of the index TAV; each of which may mean that oversizing versus the index TAV dimensions is preferred. Beneduce et al.^34^ demonstrated that, when used, oversizing versus the index TAV appeared safe and effective, whereas undersizing of the second TAV in relation to the native annulus was an independent predictor of worse clinical outcomes at 1-year post redo TAVR. Other factors that should be considered include hostile native anatomy (landing zone calcium, specifically) and the risk of coronary obstruction with overexpansion of the index TAV.

#### Second TAV Implant Depth

##### Short-in-Tall

Implant depth is predominantly determined by the risk of coronary obstruction, based on the aforementioned pre-redo TAVR CT measurements. If there is no risk, then the second TAV outflow may be implanted at the level of the top of the index TAV leaflets, avoiding leaflet overhang. If the CT indicates a potential risk of coronary obstruction, then the second TAV can be implanted deeper to reduce the NSP height below the CRP through the creation of leaflet overhang. A deeper implant may also be considered to facilitate future coronary access. Leaflet overhang should be minimized in cases of AS to avoid persistent obstruction to outflow. Notably, the long-term consequences of leaflet overhang remain unknown.

##### All Other Redo TAV Combinations

Implant inflow to inflow. A higher implant may be considered to optimize hemodynamics with tall-in-short or when the index TAV implant position was deep in relation to the native annulus. A slightly deeper implant may allow the reduction of NSP height with short-in-short.

#### Coronary Protection and/or Leaflet Modification

The risk of coronary obstruction is considered high if the valve-to-coronary distance, valve-to-STJ distance, or VTA_min_ is <2 mm and intermediate if 2-4 mm. These cutoffs are, however, based on data derived from studies of TAV-in-SAV.^28^^,^^35^ There are currently no data in relation to risk-prediction for coronary obstruction with redo TAVR.

**Figure 6:**
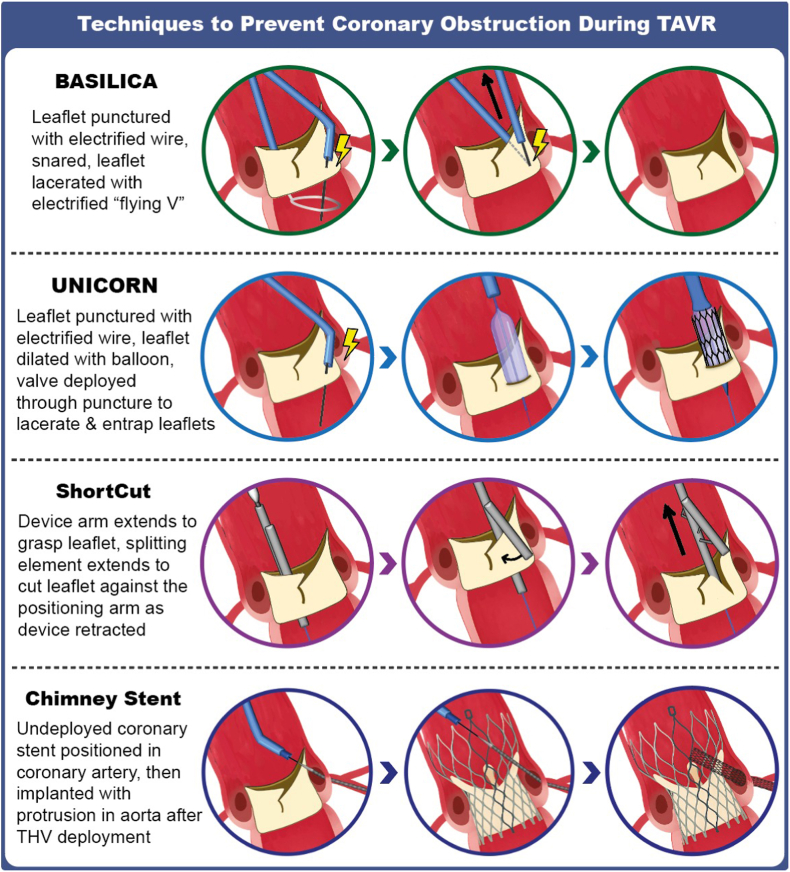
**Techniques to prevent coronary obstruction during TAVR.** Device illustrations are artistic approximations. Abbreviations: AR, aortic regurgitation; BASILICA, bioprosthetic or native aortic scallop intentional laceration to prevent iatrogenic coronary artery obstruction during TAVR; TAVR, transcatheter aortic valve replacement; THV, transcatheter heart valve; UNICORN, undermining iatrogenic coronary obstruction with radiofrequency needle.

In patients at risk of coronary obstruction, the following options should be considered and are illustrated in Figure 6:

1. Deeper implantation of a short second TAV to lower the NSP below the CRP. This is predominantly an option for short-in-tall but occasionally may be applicable with short-in-short. Bench modeling suggests that a high degree of leaflet overhang is acceptable in cases of pure regurgitation, but even with calcific stenosis of the index valve, some leaflet overhang can be achieved with effective reduction of the neoskirt height and with satisfactory hemodynamic function.^36^^,^^37^
2. Elective or provisional chimney stenting. In cases at intermediate risk—for example, VTA_min_ 2-4 mm—a provisional approach can be used by placing a guide catheter ± guide catheter extension and guidewire ± undeployed stent in the threatened coronary artery to allow stenting if needed. In high-risk cases, elective chimney stenting may be needed. Although chimney stenting is an established technique in TAV-in-SAV, bench modeling suggests that in redo TAVR, interaction with the index TAV or aortic wall may cause significant stent distortion or underexpansion.^38^ Caution is therefore required.
3. Leaflet modification

If the pre-redo TAVR CT confirms commissural and coronary alignment of the index TAV, then leaflet modification may be considered.

Bioprosthetic or native aortic scallop intentional laceration to prevent iatrogenic coronary artery obstruction (BASILICA) is the most frequently used form of leaflet modification.^39^ However, although the successful use of BASILICA has been described in redo TAVR procedures,^40^^,^^41^ bench modeling suggests that it may be less effective due to reduced splay of the lacerated leaflets, primarily as a result of the constraint created by the index TAV frame.^42^ Balloon-assisted BASILICA has also been investigated but appears to have minimal additional impact on leaflet splay in benchtop modeling.^43^^,^^44^ The undermining iatrogenic coronary obstruction with radiofrequency needle (UNICORN) procedure, in which the TAV is implanted within the leaflet that threatens the coronary artery after leaflet perforation and balloon dilatation, is likely to be more effective. Although predominantly an option with BEV as second TAV, it has also been described with SEV.^45^^,^^46^ However, further data on the optimal procedural technique, as well as the safety and efficacy of UNICORN in redo TAVR procedures, are required.

ShortCut (Pi-Cardia, Rehovot, Israel) is a dedicated, novel device designed to cut bioprosthetic aortic valve leaflets, and has demonstrated its safety and efficacy in a recently published pivotal study of patients at risk of coronary obstruction predominantly undergoing TAV-in-SAV, with 2 redo TAVR cases also included.^47^ ShortCut has been Food and Drug Administration approved since September 2024, and may represent the most promising tool for leaflet modification to facilitate redo TAVR procedures, particularly when both leaflets are treated.

### Procedural Technique

Although redo TAVR procedures may often be straightforward if appropriate preprocedural planning has been performed, benchtop modeling and early clinical experience have generated a number of tips and tricks to enhance outcomes.

#### Implant Projection

For short-in-tall procedures, the key emphasis in valve implantation is usually the position of the second TAV outflow in relation to the coronary ostia, most commonly the left coronary ostium, which is generally lower. Implantation in a left coronary cusp isolation view based on the pre-redo TAVR CT (often left anterior oblique cranial) is recommended. In all other redo TAV combinations, a projection that most favorably aligns the index and second TAVs is preferred and most commonly achieved in a right anterior oblique caudal right coronary cusp/left coronary cusp overlap view.

#### Predilatation

Predilatation should be considered if there is significant underexpansion of the index TAV. In such cases, the impact of predilatation on the VTA_min_—and thus on the risk of coronary obstruction—must be considered. Predilatation is also recommended for short-in-tall to mitigate risk of valve migration during valve deployment.

#### Valve Deployment

Both foreshortening and migration of the second TAV may occur unpredictably during TAV-in-TAV, particularly with short-in-tall combinations when the balloon-expanded second TAV interacts with the waist of the tall index TAV. Very slow deployment, with a focus on the use of the left ventricular guidewire to stabilize and control valve position, is recommended.

#### Postdilatation

Incomplete and/or eccentric expansion of the second TAV is almost inevitable after initial deployment and has been demonstrated clearly in bench models.^48^ Postdilatation, either with the original deployment balloon for BEVs or with a dedicated same-sized noncompliant balloon, is recommended in most cases. Clinical and bench data have suggested that both predilatation and postdilatation are necessary to optimize redo TAV expansion.^31^^,^^49^

## Current Evidence for Redo TAVR

**Figure 7:**
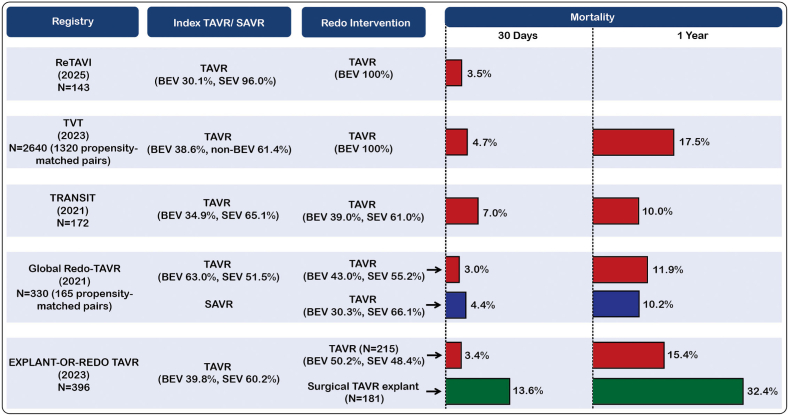
**Thirty-day and 1-year mortality after redo-TAVR—current registry data.** Abbreviations: BEV, balloon expanded valve; SAVR, surgical aortic valve replacement; SEV, self-expanding valve; TAVR, transcatheter aortic valve replacement; TAVI, transcatheter aortic valve implantation; TRANSIT, transcatheter aortic valve replacement for degenerated transcatheter aortic valves; TVT, transcatheter valve therapies.

The principal studies of redo TAVR are summarized in Figure 7. The only prospective data come from the recently presented ReTAVI registry,^50^ which included 143 patients treated with redo TAVR using a SAPIEN BEV. The most frequently treated index valves were CoreValve/Evolut (53.1%), SAPIEN (30.1%), and Acurate Neo (14.0%). The mean time to reintervention was 5.9, 7.1, and 5.6 years for the different valve types, respectively, and the TAV failure mechanism was SVD in >90% of cases. With VARC-3 device success of 95%, 30-day mortality 3.5%, stroke 0.7%, pacemaker requirement 6.3%, and ≥moderate PVR 0.9%, overall outcomes were encouraging. Incidence of coronary obstruction was only 1.4%, but notably, coronary protection was undertaken in 26.2% with 17.9% of the cohort undergoing either chimney stenting or BASILICA.

The largest multicentre retrospective registry used data from the Transcatheter Valve Therapies registry to compare 1320 propensity-matched pairs of patients undergoing redo TAVR and native TAVR using BEVs.^51^ No significant differences were observed with respect to procedural complications, death at 30 days (4.7% vs. 4.0%, *p* = 0.36) or 1 year (17.5% vs. 19.0%, *p* = 0.57), and stroke at 30 days (2.0% vs. 1.9%, *p* = 0.84) or 1 year (3.2% vs. 3.5%, *p* = 0.80).

The EXPLANT-OR-REDO study compared redo TAVR with surgical explantation plus SAVR and reported significantly higher mortality with surgery at 30 days (3.4 vs. 13.6%; *p* < 0.001) and 1 year (15.4 vs. 32.4%; *p* = 0.001).^52^

The TRANSIT registry reported outcomes up to 1 year after redo TAVR in 172 patients. Procedural success was 72%, 30-day mortality 7.0%, and 1-year mortality 10.0%.^53^

Finally, the Global Redo TAVR registry compared 165 propensity-matched pairs of patients undergoing redo TAVR and TAVR for degenerative surgical bioprostheses.^54^ Driven by lower frequency of residual high valve gradient, ectopic valve deployment, coronary obstruction, and conversion to open heart surgery, the incidence of procedural success was significantly higher with redo TAVR (72.7 vs. 62.4%; *p* < 0.05). No significant differences in 30-day mortality (3.0 vs. 4.4%; *p* = 0.57) or 1-year mortality (11.9 vs. 10.2%; *p* = 0.63) were observed.

Overall, these results have provided reassuring initial data on the outcomes of redo TAVR in selected patients. However, the only available prospective registry is a relatively small study with short-term outcomes from a single second TAV type, whereas the retrospective studies are inherently limited by selection bias and have predominantly included patients with relatively early reintervention for multiple indications including NSVD, endocarditis, and thrombosis.

## Future Studies of Redo TAVR

The REVALVE study is an investigator-initiated, multicentre European prospective observational registry that will recruit 300 patients undergoing redo TAVR with SAPIEN or Evolut for any failing index TAV.^55^ The primary endpoints are 30-day REVALVE success (adapted from VARC 3 Early safety) and 1-year freedom from mortality, stroke, and valve- or procedure-related hospitalization. There will be parallel cohorts of patients managed by surgical explantation and SAVR or by optimal medical therapy. Follow-up is for 5 years. RESTORE (ClinicalTrials.gov ID NCT06777368), sponsored by Medtronic, will recruit 225 patients in 48 centers in the United States undergoing redo TAVR with a failing index SAPIEN or Evolut valve, treated with either a SAPIEN or Evolut as the second TAV. The primary endpoints match those of REVALVE. Follow-up is for 5 years.

## Surgical Management of TAV Failure

Redo TAVR will undoubtedly be the predominant treatment for TAV BVF due to both the age and comorbidity in this patient population and the more favorable outcomes compared with surgical explantation.^50^, ^51^, ^52^, ^53^, ^54^^,^^56^, ^57^, ^58^ However, surgical management will nevertheless play an important role in specific patient groups, including those at prohibitive risk of coronary obstruction, those presenting with endocarditis, and those with either severe PPM or PVR not amenable to transcatheter intervention.

The unique challenges of explanting TAVs relate to their distinctive designs and implantation techniques, which significantly increase the risk of iatrogenic injury to the STJ, aorta, mitral valve, or membranous septum in comparison to the removal of surgical bioprostheses. In addition, concomitant disease requiring combined surgeries is frequent in this population and further increases complexity and risk. In the EXPLANT-TAVR registry, 54.6% of patients needed concomitant procedures (32.0% mitral repair/replacement and 24.5% coronary artery bypass graft), with significantly higher mortality.^56^ A lack of expertise compounds this; in the STS database from 2011 to 2018, the median number of TAV explants both per surgeon and per institution was one.

### Preoperative Planning

A preoperative CT is recommended to ensure a safe TAV explant. Several features—i.e., degree of TAV incorporation into the surrounding structures, TAV location in relation to the anatomic landmarks, and characteristics of the aortic root—suggest a more careful approach is warranted. Patients with significant adhesions should be prepared to undergo concomitant procedures: aortic root/ascending replacement for STJ involvement, mitral valve repair/replacement if the anterior mitral leaflet is impinged, or ventricular septal defect repair if it appears the membranous septum could be injured at the time of TAV explant.

### Technical Considerations

#### Cannulation, Cardioplegia, and Aortotomy

The cannulation strategy should reflect the TAV type and preoperative CT findings. For tall-frame SEVs, cannulation must be high in the aortic arch to ensure adequate room for aortic cross-clamping. Peripheral femoral or axillary cannulation should be considered if the ascending aorta is insufficient.

**Figure 8:**
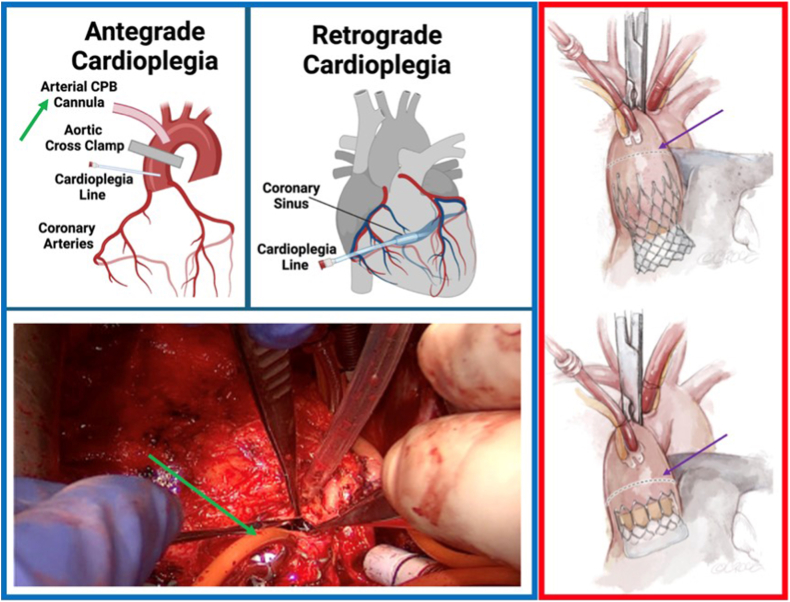
**Cardioplegia and aortotomy strategies for TAV explantation.** The left-hand panels show the two different strategies that can be used to infuse cardioplegia into the heart and stop it. The green arrow represents the site of cannulation, where the surgeon infuses the fluid to stop the heart. The right-hand panel illustrates the different TAV types and the recommended heights for aortotomy (purple arrow). Abbreviations: CPB, cardio-pulmonary bypass; TAV, transcatheter aortic valve.

Cardioplegia delivery and aortotomy also require an understanding of the TAV frame and its relationship to the coronary arteries to allow for expeditious cardioplegic arrest and exposure. If the TAV frame obstructs access to the coronary ostia, as is the case in most SEVs, then retrograde cardioplegia is the safer strategy. A retrograde approach is also typically required for initial cardiac arrest in those with AR. Otherwise, a standard antegrade approach—including after the aortotomy and TAVR explant—can be employed. (Figure 8).

The aortotomy location for BEVs is in the standard location with a transverse or oblique incision. In contrast, the aortotomy with SEVs can be performed at the top of or within the frame (Figure 8). If the frame is impalpable, an epiaortic ultrasound can be used. Occasionally, transection of the aorta is necessary and may be required for root enlargement or repair of an aortic injury.

#### Explantation of Short-Frame BEVs

##### Double Kocher Technique

**Figure 9:**
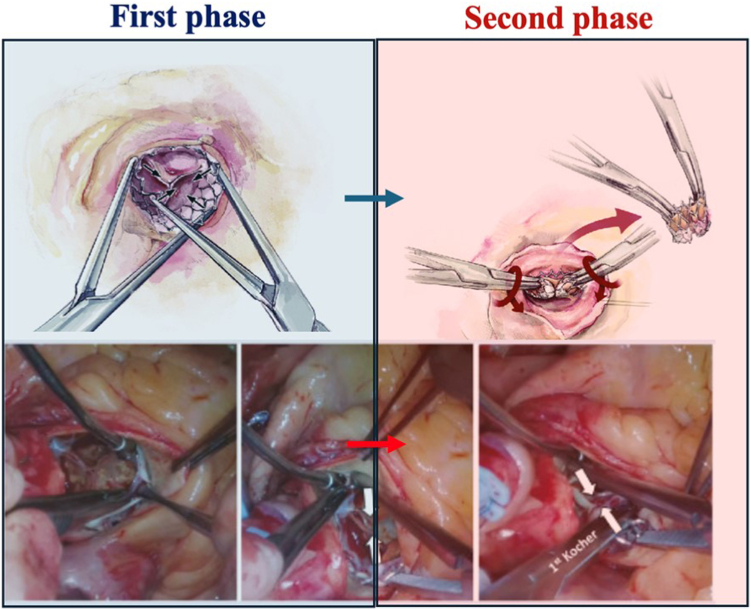
**Double Kocher technique for explantation of short-frame balloon-expanded valves.** The figure illustrates the two main phases of this technique. In the first phase, the two instruments are used to detach the prosthesis from the valve (blue box). In the second phase, the prosthesis is torqued and explanted (red box).

The fundamental technique for short-frame BEV explant is inward deformation of the stent frame. The first step is to separate the aortic wall from the distal stent frame. Typically, endarterectomy spatulas are used, but in cases with significant adhesions, a No. 11 scalpel can facilitate safe dissection. Once this is achieved circumferentially down to the halfway point of the frame, the stent is grasped with two long Kocher clamps. Perpendicular clamp application mobilizes the sharp edge of the BEV and serves as a handle for the valve explantation maneuver. As more frame is liberated, the clamps are repositioned deeper toward the base of the valve. Entering the plane between the native valve leaflets and the TAV cuff is critical. In contrast to redo SAVR, native aortic valve leaflets still exist in TAV explant, making this dissection easier if the correct plane is entered. Care must be taken with the mitral valve, left ventricular outflow tract, and membranous septum in cases with deep device implantation (Figure 9).

##### Roll Technique

**Figure 10:**
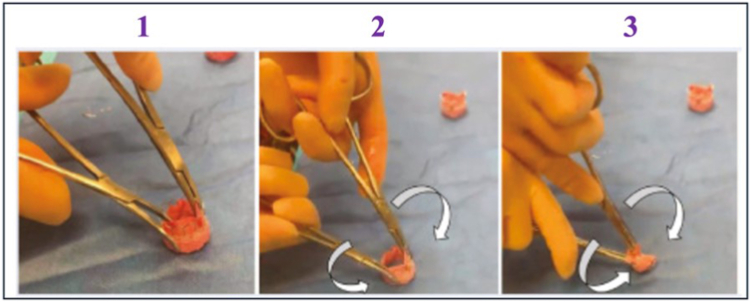
**The roll technique for explantation of short-frame balloon-expanded valves.** The figure summarizes the three main phases of this technique. The valve is rotated 360° to detach from the tissue.

First, the valve is grasped at the top of the commissures with a Tonsil or long clamp. Then, a freer elevator is used to bluntly dissect the plane between the native valve and TAV until reaching the cuff. The explant will be easier if more of the sealing cuff is freed from the aorta/aortic valve. Then, two clamps are placed 180° apart with one jaw on the inside of the BEV and the other in the plane between the native valve and BEV. The clamps are rolled inward simultaneously with a complete 360° turn on both. This collapses the valve to the smallest diameter, allowing easy prosthesis removal (Figure 10). The roll technique offers several advantages. Firstly, it requires less radial force than folding it along its diameter, so the resulting profile is much smaller along the entire length of the valve; hence, damage to the root or the aorta is less likely. Secondly, it minimizes dissection around the annulus and ascending aorta and hence decreases the risk of injury.

#### Explantation of Tall-Frame SEVs

##### Tourniquet Technique

**Figure 11:**
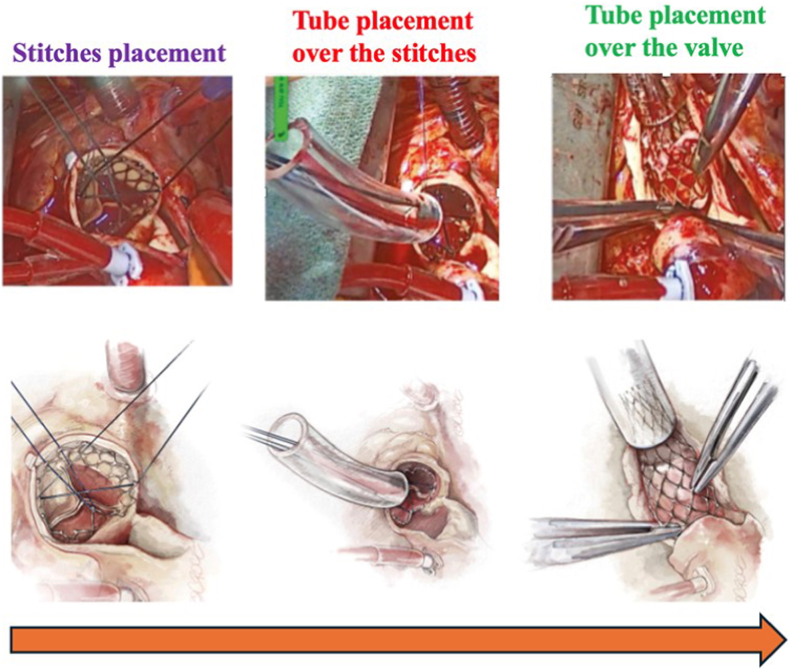
**The tourniquet technique for explantation of tall-frame self-expanding valves.** The figure illustrates the main phases of this technique. The orange arrow represents the direction of events.

Most SEVs are made of nitinol and have a tall hourglass shape, which makes dissection around the frame difficult. The Tourniquet technique can make this process safer and faster. Following the aortotomy just above the frame, a freer elevator is carefully used to lift the neointima and frame from the aortic wall. Silk ties are passed through the top cells of the frame at opposite ends then snared through a 3/8-inch pump tubing piece. The tubing is advanced to create a tourniquet and recapture the valve to reduce the profile and allow easier removal of the TAV while avoiding injury to the aorta from the top of the frame (Figure 11).

##### Handlebar Moustache Technique

Radial infolding of SEVs, the so-called handlebar moustache technique, has also been described; the valve is divided in half transversely to remove the crown and then cut longitudinally. The valve is then grasped at the cut edges and enfolded. Although there are concerns regarding the sharp edges of the metal frame, it is an alternative to the tourniquet technique.

#### Early Versus Late TAV Explant

The early TAV explant will have minimal adhesions, which makes dissection of the stent frame easier. The early TAV explant will occur in patients with endocarditis, PVR, PPM, or early SVD. The late TAV explant will have significant endothelialization covering the TAV, which will make dissection difficult. As the TAV explant population moves to low-risk young patients and early failure is less common, late TAV failure due to SVD will become the predominant pathology. Current TAV explant data are dominated by early failure, with evidence on TAV explant after 5–10 years or more minimal. Meticulous dissection using a scalpel to prevent injury to the aortic root structures and to avoid unnecessary aortic root replacement will be necessary in these patients, and procedural complexity and risk may be higher still.

## Specific Prosthesis Types

In common with transcatheter treatment, an understanding of the index TAV design is critical to surgical explantation. Several TAV types have unique design features that affect explant technique.

### Acurate Neo

The valve has stabilization arches at the outflow and a conventional inflow similar to other tall SEV devices. However, the upper crown in the midportion is 5 mm larger in diameter than the main body of the valve, and due to its flared nature, the tourniquet technique cannot be used. Extra care is required to prevent injury to the aorta during its removal once it is entirely freed from the annulus and removed sideways.

### Lotus (Boston Scientific, Minneapolis, Minnesota)

This short, intra-annular, mechanically expanded prosthesis has commissural posts with a unique “seat buckle” deployment and locking mechanism. Manual unbuckling of the posts can be accomplished by pressing the buckle, which facilitates removal and allows the valve to stretch. The double Kocher or Roll technique can be used if accessing the buckle is impossible due to tissue ingrowth. The valve height is 19 mm across all sizes and in small anatomy may sit above the STJ; this makes removal more challenging.

## Combined Procedures

### Aortic Root Enlargement

In the setting of PPM, aortic root enlargement may be necessary; the need for which can be predicted from the preoperative CT. Different aortic enlargement techniques can be used and will not be discussed in detail here.

### Aortic Root Replacement

In the STS database, roughly 20% of TAV explants required aortic root replacement regardless of the explanted TAV type.^58^ Particularly when associated with a periannular abscess, root replacement is most frequent in the setting of endocarditis. It may also be required when explanting a TAV that has deeply endothelialized into the native aortic wall and thereby causes damage to the proximal ascending aorta or sinuses at the time of explant. Less often, root replacement is needed to address a root aneurysm, aortic dissection, pseudoaneurysm, or TAV degeneration in a small aortic root. Despite added complexity, in experienced hands, root replacement can be performed with similar periprocedural outcomes as SAVR for TAV explant.

### Mitral Valve Surgery With Previous TAVR

Mitral valve surgery is frequently required during TAV explant and carried out in a standard fashion after explantation. A deeply implanted TAV may be encased with the mitral valve anterior leaflet and require careful dissection to avoid injury to the aortomitral curtain (AMC). For endocarditis extending into the AMC, extensive reconstruction or a Commando operation may be necessary.

## Alternative Technique (Surgical Resection of Prosthetic Valve Leaflets Under Direct Vision)

SUrgical Resection of Prosthetic Valve Leaflets Under Direct ViSion (SURPLUS) is a hybrid technique combining surgical resection of the TAV leaflets with implantation of a BEV under direct vision and fluoroscopic guidance.^59^ After initiation of cardiopulmonary bypass, aortic cross-clamp, and cardioplegia, an oblique aortotomy is performed above the frame. The TAV leaflets are resected, but the frame is left intact. Under direct vision, a BEV is positioned over a guidewire, and the valve commissures are aligned. Using fluoroscopic guidance, the valve is deployed slowly at its intended position.

SURPLUS avoids tissue dissection during TAV explant, preventing aortic or other structural injuries. Its shortening of the cardiopulmonary bypass and aortic cross-clamp times may be critical for high-risk patients. It represents an alternative option in patients where redo TAVR is not possible due to a prohibitive risk of coronary obstruction, but it should not be used where TAV BVF is due to endocarditis, PVR, or PPM.

## Surgery for TAV Endocarditis

TAV infective endocarditis (IE) is relatively infrequent but carries a disproportionate burden of embolism, conduction system involvement, and periannular extension.

Surgical indications mirror those for prosthetic IE in general.^13^^,^^26^

Technically, TAV IE almost always demands radical debridement. After establishing protection (often favoring addition of retrograde cardioplegia when the root is compromised), the infected TAV and all devitalized tissues are removed. The operation then becomes one of reconstruction: annular abscess cavities are saucerized to healthy margins and obliterated with pericardial or Dacron patches; AMC defects are repaired or replaced with single- or double-patch techniques; and, when destruction is circumferential or involves the entire root, a composite graft root replacement with coronary reimplantation is performed.^13^^,^^26^^,^^60^ In extensive AMC destruction or aortoventricular discontinuity, a Commando-type reconstruction (patch reconstruction of the AMC combined with aortic and mitral valve replacement) restores geometry at the cost of longer bypass and cross-clamp times.^60^

### Pitfalls Are Predictable

Annular and root tears during extraction of a frame tethered to inflamed tissue, stroke from mobilization of vegetations and friable debris, and postoperative conduction block when the abscess extends toward the membranous septum. Preparation with patches, felt buttresses, and root grafts alongside meticulous debridement and systematic evacuation of thrombocalcific debris mitigate these hazards.^13^^,^^26^^,^^60^

## Outcomes of Surgery for TAV BVF

In the EXPLANT-TAVR International Registry (n = 269, 2009–2020), the median time to explantation was 11.5 months; endocarditis (43%), SVD (20%), paravalvular leak (18%), and PPM (11%) were the leading indications. In-hospital, 30-day, and 1-year mortality were 12%, 13%, and 29%, respectively, with stroke at 9% at 30 days.^58^

Other series corroborate substantial early hazard, especially in urgent or emergent cases, operations requiring root replacement, and endocarditis.^56^, ^57^, ^58^^,^^61^ Despite this early risk, conditional survivors often achieve acceptable mid-term survival, supporting surgery when anatomy or infection renders transcatheter options unsafe or nondefinitive.^56^, ^57^, ^58^

A recent national analysis reported 1,346 TAV explants among >410,000 index TAVRs—rising from 0.17% (2019) to 0.28% (2023) of cumulative cases—illustrating that although absolute numbers remain modest, TAV explant is one of the fastest-growing operations in contemporary cardiac surgery and benefits from concentration at experienced centers.^6^

## Conclusions

The management of TAV failure will become an increasingly frequent challenge for both interventional cardiologists and cardiac surgeons. An awareness of the key issues and the optimal strategies for diagnosis, preprocedural planning, and procedural execution is critical to deliver safe and effective treatment for this growing patient population.

## Funding

The authors have no funding to report.

## Disclosure Statement

Daniel J Blackman reports a relationship with Abbott Vascular Inc that includes consulting or advisory and speaking and lecture fees; Edwards Lifesciences Corporation that includes consulting or advisory; JenaValve Technologies that includes consulting or advisory and speaking and lecture fees; and Medtronic that includes consulting or advisory, funding grants, and speaking and lecture fees. Noman Ali reports a relationship with Abbott Vascular Inc that includes consulting or advisory and speaking and lecture fees, Edwards Lifesciences Corporation that includes consulting or advisory and speaking and lecture fees; and Medtronic Inc that includes consulting or advisory and speaking and lecture fees. Mariama Akodad reports a relationship with Abbott Vascular Inc that includes consulting or advisory and speaking and lecture fees and Medtronic that includes consulting or advisory and speaking and lecture fees. Annette Maznyczka reports a relationship with Abbott Vascular Inc that includes travel reimbursement, Boston Scientific Corporation that includes travel reimbursement, Edwards Lifesciences Corporation that includes travel reimbursement, and Medtronic that includes travel reimbursement. David Hildick-Smith reports a relationship with Abbott Vascular Inc that includes consulting or advisory and speaking and lecture fees, Boston Scientific Corporation that includes consulting or advisory and speaking and lecture fees, Edwards Lifesciences Corporation that includes consulting or advisory and speaking and lecture fees, Meril Life Sciences Private Limited that includes consulting or advisory and speaking and lecture fees, and Medtronic that includes consulting or advisory and speaking and lecture fees. Edoardo Zancannaro reports a relationship with Edwards Lifesciences Corporation that includes consulting or advisory and speaking and lecture fees. Hendrik Treede reports a relationship with Edwards Lifesciences Corporation that includes consulting or advisory and speaking and lecture fees, JenaValve Technology Inc that includes consulting or advisory and speaking and lecture fees, and Medtronic that includes consulting or advisory and speaking and lecture fees.
